# Early clinical outcomes of transcatheter aortic valve implantation using the NAVITOR system

**DOI:** 10.1007/s12928-024-01081-7

**Published:** 2025-01-06

**Authors:** Kosuke Fujita, Koichiro Matsumura, Keishiro Sugimoto, Kyohei Onishi, Kazuyoshi Kakehi, Ayano Yoshida, Takayuki Kawamura, Masakazu Yasuda, Hiroki Matsuzoe, Kazuki Mizutani, Tatsuya Miyoshi, Masafumi Ueno, Genichi Sakaguchi, Gaku Nakazawa

**Affiliations:** 1 Division of Cardiology, Department of Medicine, Kindai University Faculty of Medicine, 377-2 Ohno-Higashi, Osakasayama, Osaka 589-8511 Japan; 2 Division of Cardiology, Sakurabashi Watanabe Advanced Healthcare Hospital, Osaka, Japan; 3 Division of Cardiology, Sapporo Cardio Vascular Clinic, Sapporo, Japan; 4 Division of Cardiovascular Surgery, Department of Surgery, Kindai University Faculty of Medicine, Osakasayama, Japan

**Keywords:** Aortic stenosis, Transcatheter aortic valve implantation, NAVITOR system, Hypo-attenuated leaflet thickening

## Abstract

Transcatheter aortic valve implantation (TAVI) using the NAVITOR system has been relatively underreported due to its recent introduction in Japan. This study aimed to assess the short-term outcomes of TAVI with the NAVITOR in real-world clinical practice. Patients with severe aortic stenosis who underwent TAVI using the NAVITOR system at our institution between December 2022 and December 2023 were prospectively enrolled. We evaluated the 30-day post-TAVI outcomes using the Valve Academic Research Consortium 3 (VARC-3). Among 32 consecutive patients (mean age, 84 years; 19% female), technical success was achieved in 31 (96.8%). One patient developed a subclavian artery vascular dissection, requiring balloon angioplasty during the TAVI procedure, and another received a permanent pacemaker due to persistent advanced atrioventricular block. At the 30-day follow-up, device success was achieved in 31 (96.8%) patients, with early safety observed in 27 (84.3%). One patient experienced sudden unexplained death after discharge. None of the patients experienced myocardial infarction, valve embolisation, life-threatening bleeding, or acute kidney injury within 30 days after the procedure. Echocardiographic follow-up showed a median effective orifice area index of 1.26 ± 0.27. Contrast-enhanced computed tomography was performed in 19 patients 30 days after the procedure, revealing hypo-attenuated leaflet thickening (HALT) in 10 (52.6%). Although the 30-day outcomes of TAVI using the NAVITOR system appeared promising, a relatively high incidence of HALT was observed.

**Graphical Abstract:**

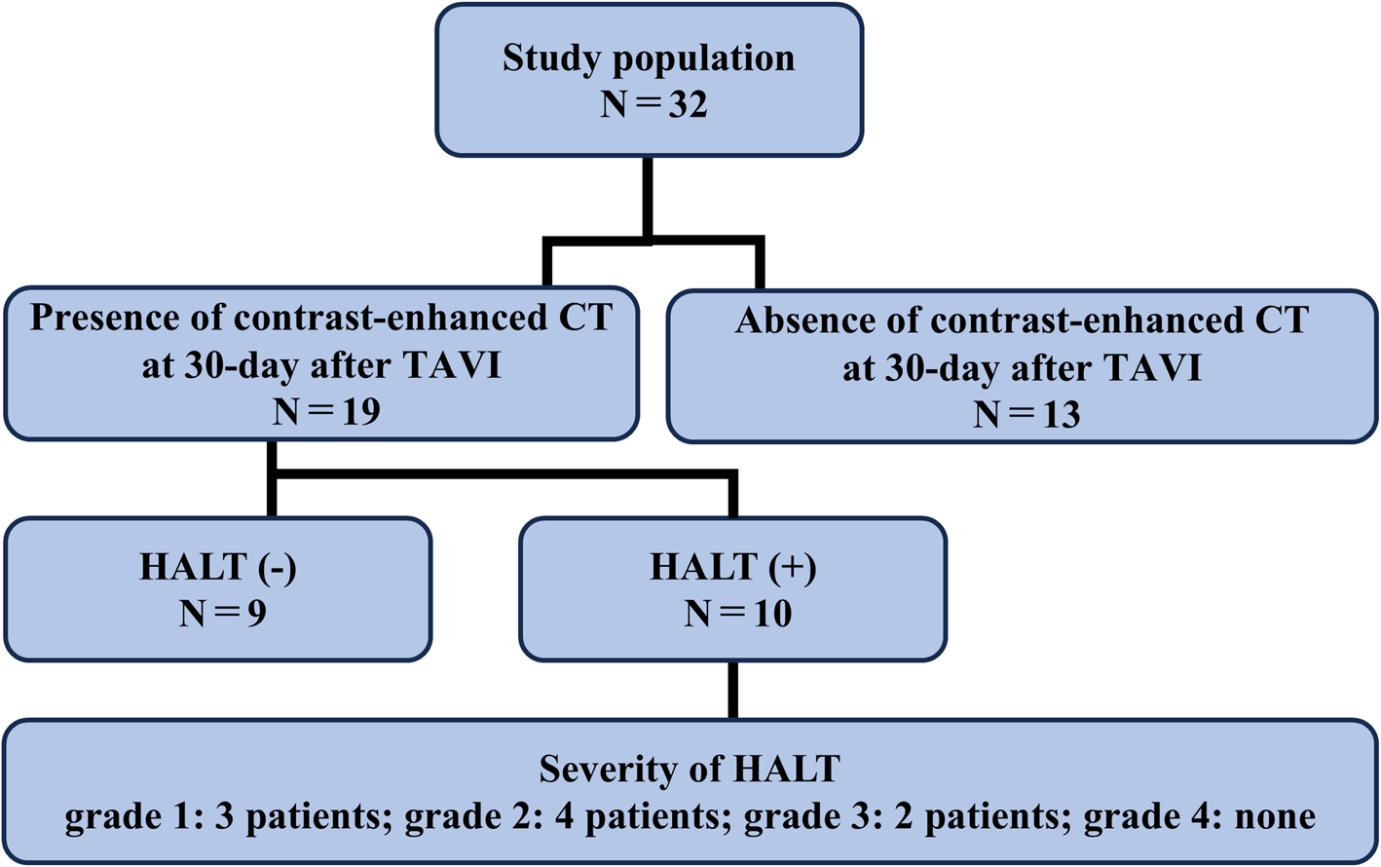

## Introduction

Transcatheter aortic valve implantation (TAVI) is an effective treatment for severe aortic stenosis (AS) and is recommended for elderly patients across all surgical risk categories in Europe, the United States, and Japan; however, indications for TAVI have expanded to lower risk patients and younger age groups [1–3]. Key considerations for the lifelong management of these patients include the haemodynamic performance of the valve design, prevention of paravalvular leak (PVL), and ensuring coronary artery access for future percutaneous coronary interventions.

The self-expanding valve NAVITOR system manufactured by Abbott has emerged as a new concept for TAVI treatment [4]. The NAVITOR valve has intra-annular leaflets and large frame cells, achieving smooth coronary artery access for future coronary artery intervention after TAVI value deployment. In addition, the NAVITOR valve also incorporates a high outer fabric cuff designed to reduce PVL by synchronising with the cardiac cycle, expanding to fill the gaps caused by calcification between the aortic annulus and the prosthesis [5]. Although excellent clinical outcomes have been demonstrated with the NAVITOR system in Europe and the United States, a high pacemaker implantation rate has been identified as a potential disadvantage [5]. In Japan, favourable clinical outcomes have been observed in terms of haemodynamics and reduced PVL, particularly in cases involving a small annulus [6]. However, few studies have focussed on valve deployment techniques, positioning, or postoperative computed tomography (CT) findings. This study, therefore, aims to evaluate the 30-day feasibility and safety of TAVI using the NAVITOR system, with a focus on procedural methods and postoperative CT assessments.

## Methods

### Study design

Between December 2022 and December 2023, 32 consecutive patients who underwent TAVI using the NAVITOR system at Kindai University Hospital in Japan were prospectively enrolled. The sole inclusion criterion was any patient undergoing TAVI with the NAVITOR system at the hospital during this period. No specific exclusion criteria were applied. The procedure was performed either via the transfemoral or alternative access routes. This study was approved by the Ethics Committee of Kindai University Hospital (R03-040), and written informed consent was obtained from all the patients.

### Study device

**Fig. 1:**
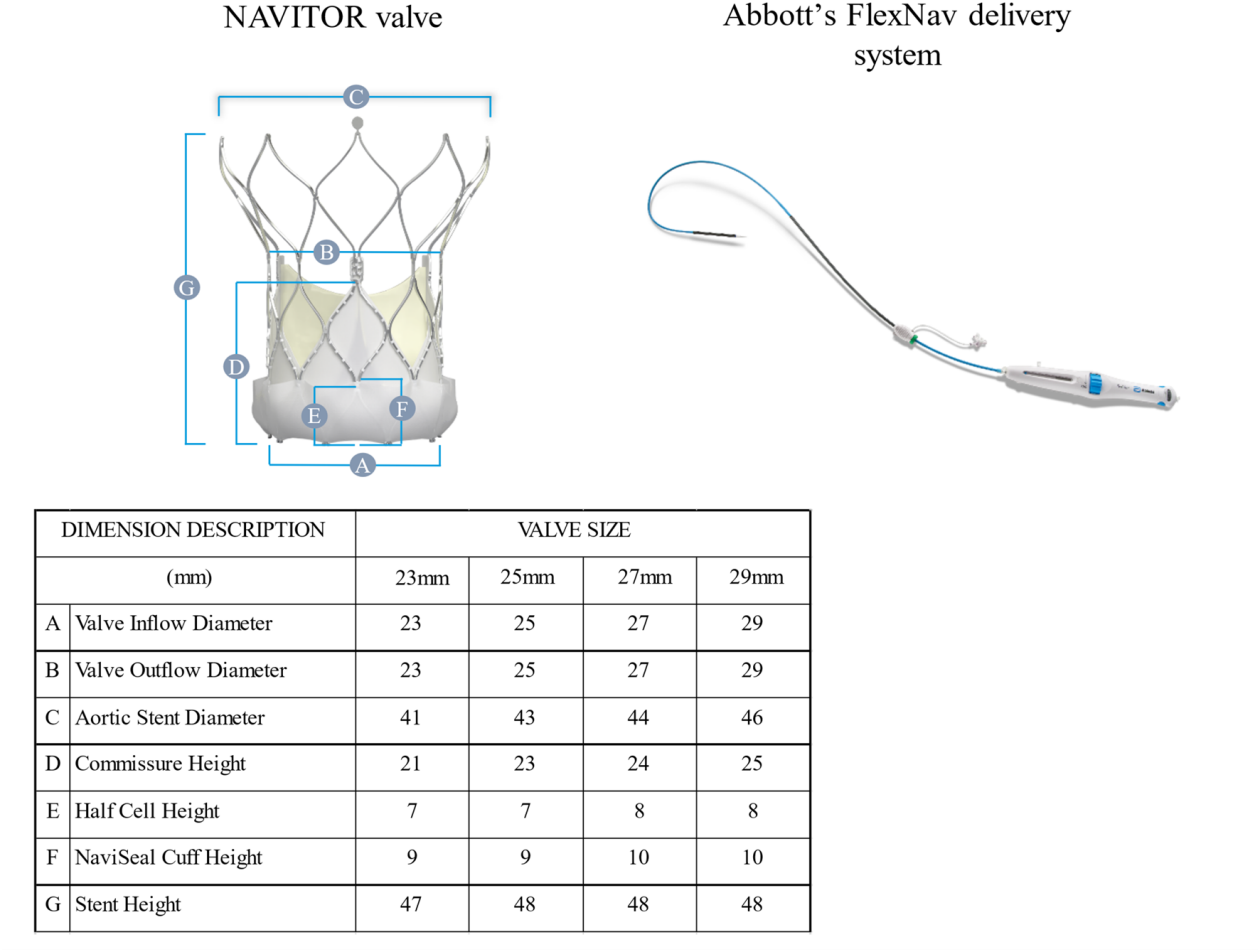
Components of the NAVITOR transcatheter aortic valve implantation system and dimensions of the NAVITOR valve. The NAVITOR valve (upper left) and the delivery system (upper right). Labelled A through G, the dimensions for each valve size are shown (lower). Information is cited from the NAVITOR instruction for use (lower right)

The NAVITOR system is a repositionable, self-expanding valve featuring a non-tapered stent designed to minimise interaction with the heart’s conduction system. It has large stent cells to facilitate future coronary access for interventions and includes three intra-annularly positioned bovine pericardial tissue leaflets. The NAVITOR valve is available in four sizes (23, 25, 27, and 29 mm), accommodating an aortic annulus ranging from 19 to 27 mm. Key design features of the NAVITOR valve include an active NaviSeal cuff on the exterior portion of the stent to optimise valve sealing and reduce PVL, a more uniform chronic outward radial force across all valve sizes, and minor modifications to the stent design aimed at minimising vessel trauma. The height of the NaviSeal cuff is 9 mm for the 23 and 25 mm valves and 10 mm for the 27 and 29 mm valves (Fig. 1). To optimise valve sealing and minimise the risk of conduction disturbances post-implantation, the target implant depth for the NAVITOR valve is 3 mm. The NAVITOR valve is compatible with the FlexNav delivery system, which is designed to offer enhanced flexibility for deliverability and stable positioning during deployment. The 23 and 25 mm NAVITOR valve can be implanted using the 14 Fr equivalent FlexNav delivery system in access vessels with a diameter of ≥ 5.0 mm, whereas the 27 and 29 mm NAVITOR valve can be implanted using the 15 Fr equivalent large FlexNav delivery system in access vessels with a diameter of ≥ 5.5 mm.

### TAVI procedure

The indication for TAVI treatment was determined by a consensus from our hospital’s heart team, which included cardiologists, cardiovascular surgeons, and other healthcare professionals. Patients were included if they presented with severe symptomatic AS and were considered to be at high or extreme risk. Severe AS was defined as an aortic valve area (AVA) of < 1.0 cm^2^ or an AVA indexed to body surface area (BSA) of < 0.6 cm^2^/m^2^. Severe AS was categorised into a high-pressure gradient type, with a peak transvalvular velocity of ≥ 4.0 m/s and a mean pressure gradient of ≥ 40 mm Hg, and a low-pressure gradient type, with a peak velocity of < 4.0 m/s and a mean pressure gradient of < 40 mm Hg. Low-flow/low-gradient severe AS was further divided into two types: low stroke volume with reduced left ventricular ejection fraction (LVEF) and preserved LVEF with a small left ventricular cavity. An independent screening committee of physician investigators reviewed all patients to confirm eligibility, surgical risk, and anatomical suitability. In addition, all patients underwent contrast-enhanced CT before the procedure for proper device sizing and procedural planning. Valve implantation was performed under general or local anaesthesia, with the access site prepared according to standard protocols. Balloon aortic valvuloplasty was recommended for pre-dilation of the native aortic valve according to the manufacturer’s instructions. The operator could choose to use the FlexNav delivery system integrated sheath alone or with an external introducer sheath to deploy the NAVITOR valve. During valve deployment, the aim was to achieve an implant depth within 3 mm of the non-coronary cusp using the cusp-overlap view, avoiding the distal opening method [7]. If the implantation depth or positioning was suboptimal, valve re-sheathing and repositioning were performed before full release. According to the manufacturer’s instructions, the valve could be re-sheathed and repositioned before complete release. Post-dilation was performed at the operator’s discretion to enhance sealing in cases of clinically significant PVL or valve under expansion. Post-procedural antithrombotic therapy was administered according to standard care protocols.

### Data collection

Patient medical history, baseline laboratory parameters, and baseline electrocardiogram data were collected. Echocardiography was performed by a cardiologist at baseline, immediately after the procedure, and 30 days after the procedure. Clinical events, including mortality, stroke, myocardial infarction, bleeding, access-site complications, permanent pacemaker implantation, and the New York Heart Association (NYHA) class, were evaluated according to the VARC-3 definitions [8]. Pre-procedural contrast-enhanced CT was used to determine valve size and evaluate the membranous septum (MS) distance. The MS was measured using the 3mensio Structural Heart software program (Pie Medical Imaging, Bilthoven, Netherlands). For standardised analysis, the cursor in the perpendicular co-planar view was positioned at the intersection of the non-coronary and right coronary cusps. MS was defined in this view as the thinnest part of the interventricular septum between the left ventricular outflow tract and the right atrium, extending from the nadir of the non-coronary cusp to the tip of the muscular interventricular septum, often delineated by the hinge point of the septal leaflet of the tricuspid valve [9]. At 30 days post-procedure, contrast-enhanced CT was performed to evaluate hypo-attenuated leaflet thickening (HALT), except in patients with renal dysfunction or other contraindications. HALT was defined as a hypo-attenuated thickening, with or without leaflet rigidity, visible in at least two different multiplanar reformation projections and two different reconstruction time intervals [10, 11]. The HALT severity was graded per leaflet, using a 4-tier grading scale based on leaflet involvement along the curvilinear contour, assuming maximum involvement at the base of the leaflet: grade 1, ≤ 25% (limited to the base); grade 2, > 25% and ≤ 50%; grade 3, > 50% and ≤ 75%; grade 4, > 75% [12]. Antiplatelet or anticoagulation therapy prescribed at discharge was also documented.

### Statistical analysis

Continuous variables were presented as the mean ± standard deviation or median (interquartile range) and categorical variables were presented as frequencies and percentages, as appropriate. A *t*-test was conducted to compare the mean effective orifice area, mean effective orifice area index, peak aortic valve gradient, and mean aortic valve gradient between the groups with and without HALT, based on echocardiographic findings 30 days after the procedure. Statistical significance was set at *p* < 0.05. All statistical analyses were performed using JMP Pro 16.2.0 (SAS Institute Inc., Cary, NC, USA).

## Results

Among the 32 patients who underwent TAVI with the NAVITOR system, the mean age was 84.5 ± 4.6 years, and 26 (81.2%) were female. The clinical frailty score averaged 4.0 ± 0.7, and the Society of Thoracic Surgeons (STS) score was 6.0 (IQR 3.8–9.0) (Table 1). At baseline, seven patients were classified as NYHA III or IV. The mean LVEF was 65.8 ± 7.3%, the mean aortic valve orifice area was 0.75 ± 0.20 cm^2^, and the mean pressure gradient was 40.9 ± 14.9 mm Hg before the procedure (Table 2). None of the patients had right or left bundle branch block. Pre-procedural CT indicated a mean annulus area of 380.4 ± 58.8 mm^2^ and a mean annulus perimeter of 71.6 ± 5.5 mm (Table 2). The mean MS length was 4.9 ± 1.3 mm.

**Table 1:** Baseline characteristics

Baseline patient characteristics	*N* = 32
Age (years)	84.5 ± 4.6
Female	26 (81)
Height (cm)	148 ± 9
Weight (kg)	48.3 ± 9.3
Body mass index (kg/m^2^)	21.9 ± 3.3
Body surface area (m^2^)	1.39 ± 0.15
Clinical frailty score	4.0 ± 0.7
STS score	6.0 (3.8—9.0)
NYHA functional class III or IV	7 (21)
Arteriosclerosis risk	
Hypertension	29 (91)
Diabetes mellitus	8 (25)
Dyslipidaemia	16 (50)
Smoking current or former	8 (25)
Previous illness	
Atrial fibrillation	7 (22)
Permanent pacemaker implanted	1 (3)
Myocardial infarction	2 (6)
Coronary stenting	8 (25)
Coronary artery bypass graft	1 (3)
Peripheral vascular disease	4 (13)
Stroke	3 (9)
Chronic obstructive pulmonary disease	2 (6)
Laboratory data	
Haemoglobin (g/dL)	11.5 ± 1.5
Haematocrit (%)	35 ± 5
Creatinine (mg/dL)	0.8 (0.7—1.2)
Albumin (g/dL)	3.6 ± 0.6
Brain natriuretic peptide (pg/mL)	158 (90—463)

Values are mean ± standard deviation or values are median (interquartile range) or *n* (%)

*NYHA* New York Heart Association, *STS* Society of Thoracic Surgeons

**Table 2:** Preoperative parameters

Echocardiography	*N* = 32
Left ventricle ejection fraction (%)	65.8 ± 7.3
Peak aortic velocity (m/s)	4.1 ± 0.5
Aortic valve area (cm^2^)	0.75 ± 0.20
Index aortic valve area (cm^2^/m^2^)	0.54 ± 0.15
Peak aortic valve pressure gradient (mmHg)	70.1 ± 18.7
Mean aortic valve pressure gradient (mmHg)	40.9 ± 14.9
Moderate or severe aortic regurgitation	6 (19)
Moderate or severe mitral regurgitation	5 (16)
Moderate or severe tricuspid regurgitation	4 (13)
Systolic pulmonary artery pressure (mmHg)	32.0 ± 9.4
Bicuspid aortic valve disease	1 (3)
CT	
Annulus area (mm^2^)	380.4 ± 58.8
Annular perimeter (mm)	71.6 ± 5.5
Annular mean diameter (mm)	21.9 ± 1.6
MS length (mm)	4.9 ± 1.3
ECG	
Left bundle branch block	0 (0)
Right bundle branch block	0 (0)
Pacemaker waveform	1 (3)

Values are mean ± standard deviation or *n* (%)

*CT* computed tomography, *ECG* electrocardiogram, *MS* membrane septum

During the procedure, 25 (78.1%) patients were treated under local anaesthesia with sedation (Table 3). TAVI was performed via the femoral artery in 28 (88%) patients. The sizes of the implanted NAVITOR valves were 23 mm in seven, 25 mm in 18, 27 mm in five, and 29 mm in two patients. The mean oversizing rate of the NAVITOR valves was 10.2%. The mean implant depth, estimated by aorta gram after valve implantation, was 3.1 ± 1.8 mm from the non-coronary cusp and 4.7 ± 1.9 mm from the left-coronary cusp. In five patients, the implant depth from the non-coronary cusp exceeded the MS length. In the majority of cases, the MS length was longer than the implant depth of the non-coronary cusp, and the mean value of MS length–implant depth of the non-coronary cusp was 1.75 ± 2.0 mm (Table 3).

**Table 3:** Procedural characteristics

Procedural characteristics	*N* = 32
Type of anaesthesia	
Local anaesthesia with sedation	25 (78)
Conversion	2 (6)
Volume of contrast (cc)	89 ± 33
Access route	
Trans femoral	28 (88)
Trans subclavian	4 (13)
Pre-balloon valvuloplasty	32 (100)
Coronary protection	1 (3)
Device size	
23 mm	7 (22)
25 mm	18 (56)
27 mm	5 (16)
29 mm	2 (6)
Oversizing rate* (%)	10.2 ± 4.1
Recapture	14 (43)
Once	6 (18)
Twice	4 (12)
Three times	4 (12)
Recapture (count)	0.81 ± 1.0
ID NCC (mm)	3.1 ± 1.8
ID LCC (mm)	4.7 ± 1.9
MS-ID NCC (mm)	1.75 ± 2.0
Post-balloon valvuloplasty	2 (6)
Technical success	31 (97)
Procedural success	32 (100)
Hospital stay (days)	7.5 (6.0—12.2)

Values are mean ± standard deviation or values are median (interquartile range) or *n* (%)

*ID* implant depth, *NCC* non-coronary cusp, *LCC* left-coronary cusp, *MS* membranous septum, *CT* computed tomography

^*^Oversizing rate was calculated using the formula: (device nominal perimeter/CT-derived annular perimeter-1) × 100

A total of 14 (43.7%) patients underwent recapture: 6 (18.7%) patients required a single recapture, 4 (12.5%) patients underwent two recaptures, and 4 (12.5%) patients underwent three recaptures. The mean count of recaptures was 0.8 ± 1.0 (Table 3).

Technical success was achieved in 31 (96.8%) patients, with a mean hospital stay of 10.8 ± 7.5 days.

Although all patients successfully received the NAVITOR valve, one (3.1%) required balloon dilation for vascular dissection of the subclavian artery that occurred during device delivery. In addition, new-onset complete left bundle branch block was observed in two (6.2%) following TAVI, with one (3.1%) requiring permanent pacemaker implantation. No other hospital events were reported (Table 4). At 30 days after the procedure, the device success rate was 96.8% (31 patients), and early safety was 90.6% (29 patients). One patient experienced sudden unexplained death after discharge. None of the patients had myocardial infarction, valve embolisation, life-threatening bleeding, or acute kidney injury during the 30-day follow-up.

**Table 4:** Clinical outcomes and echocardiographic parameters after the procedure

30-day clinical outcomes	*N* = 32
Device success	31 (97)
Early safety	29 (91)
Adverse events	
Death	1 (3)
Stroke	0 (0)
Bleeding	0 (0)
Access-site complication	1 (3)
Permanent pacemaker implantation	1 (3)
Rehospitalisation	0 (0)
Echocardiography immediately after the procedure	
Peak aortic valve gradient (mmHg)	15.2 ± 5.1
Mean aortic valve gradient (mmHg)	7.9 ± 3.3
Valve effective orifice area (cm^2^)	1.78 ± 0.33
Valve effective orifice area index (cm^2^/m^2^)	1.28 ± 0.28
Aortic regurgitation	
None/trivial	26 (81)
Mild	6 (19)
Moderate	0 (0)
Severe	0 (0)
Mitral regurgitation	
None/trivial	20 (63)
Mild	8 (25)
Moderate	4 (13)
Severe	0 (0)
Systolic pulmonary artery pressure (mmHg)	31.3 ± 8.0
Echocardiography 30 days after the procedure	
Peak aortic valve gradient (mmHg)	14.4 ± 5.2
Mean aortic valve gradient (mmHg)	7.2 ± 5.1
Valve effective orifice area (cm^2^)	1.78 ± 0.27
Valve effective orifice area index (cm^2^/m^2^)	1.27 ± 0.22
Aortic regurgitation	
None/trivial	25 (92)
Mild	2 (7)
Moderate	0 (0)
Severe	0 (0)
Mitral regurgitation	
None/trivial	16 (59)
Mild	10 (37)
Moderate	1 (4)
Severe	0 (0)
Systolic pulmonary artery pressure (mmHg)	31.7 ± 8.3

Values are mean ± standard deviation or *n* (%)

Echocardiography immediately after the procedure revealed a mean effective orifice area index of 1.26 ± 0.27 cm^2^/m^2^. Moderate or severe prosthesis-patient mismatch was observed in one patient, who had an effective orifice area index of 0.79 cm^2^/m^2^. Furthermore, none of the patients exhibited moderate or severe aortic regurgitation; however, mild regurgitation was present in five (18.7%) patients. At the 30-day follow-up, the mean effective orifice area index was 1.27 ± 0.22 cm^2^/m^2^ (Table 4). None of the patients had moderate or severe prosthesis-patient mismatch at this time. The patient with moderate prosthesis-patient mismatch immediately after the procedure showed improvement, with the effective orifice area index expanding to 0.97 at 30 days. Furthermore, none of the patients had moderate or severe aortic regurgitation, with mild regurgitation occurring in two (7.4%) patients (Table 4).

**Fig. 2:**
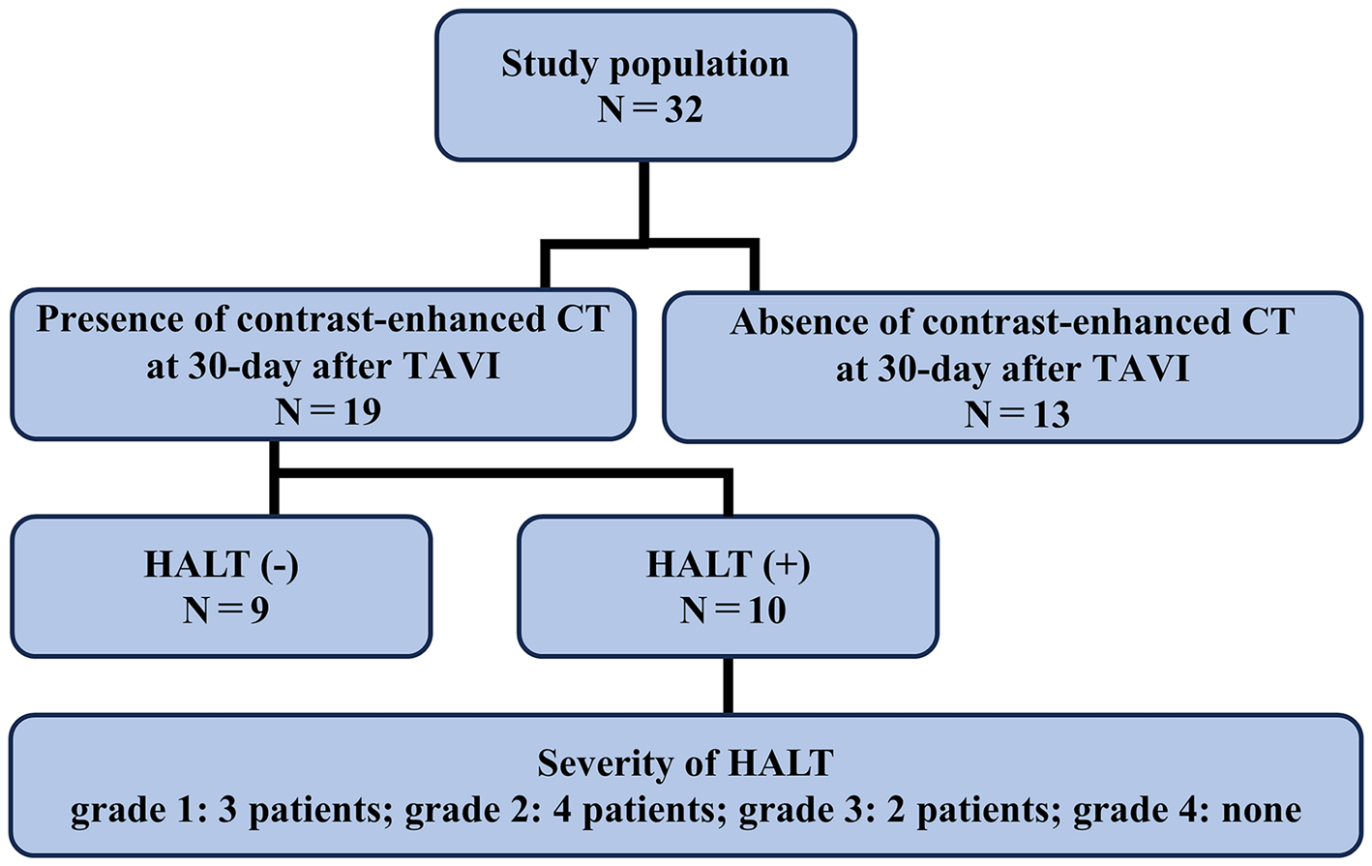
The presence and degree of HALT on contrast-enhanced CT 30 days after the procedure. Contrast-enhanced CT was performed 30 days after TAVI on 19 of 32 cases, with HALT observed in 10 cases. The severity of HALT was graded as follows: grade 1 in three, grade 2 in four, and grade 3 in three cases. *HALT* hypo-attenuated leaflet thickening, *TAVI* transcatheter aortic valve implantation, *CT* computed tomography

**Fig. 3:**
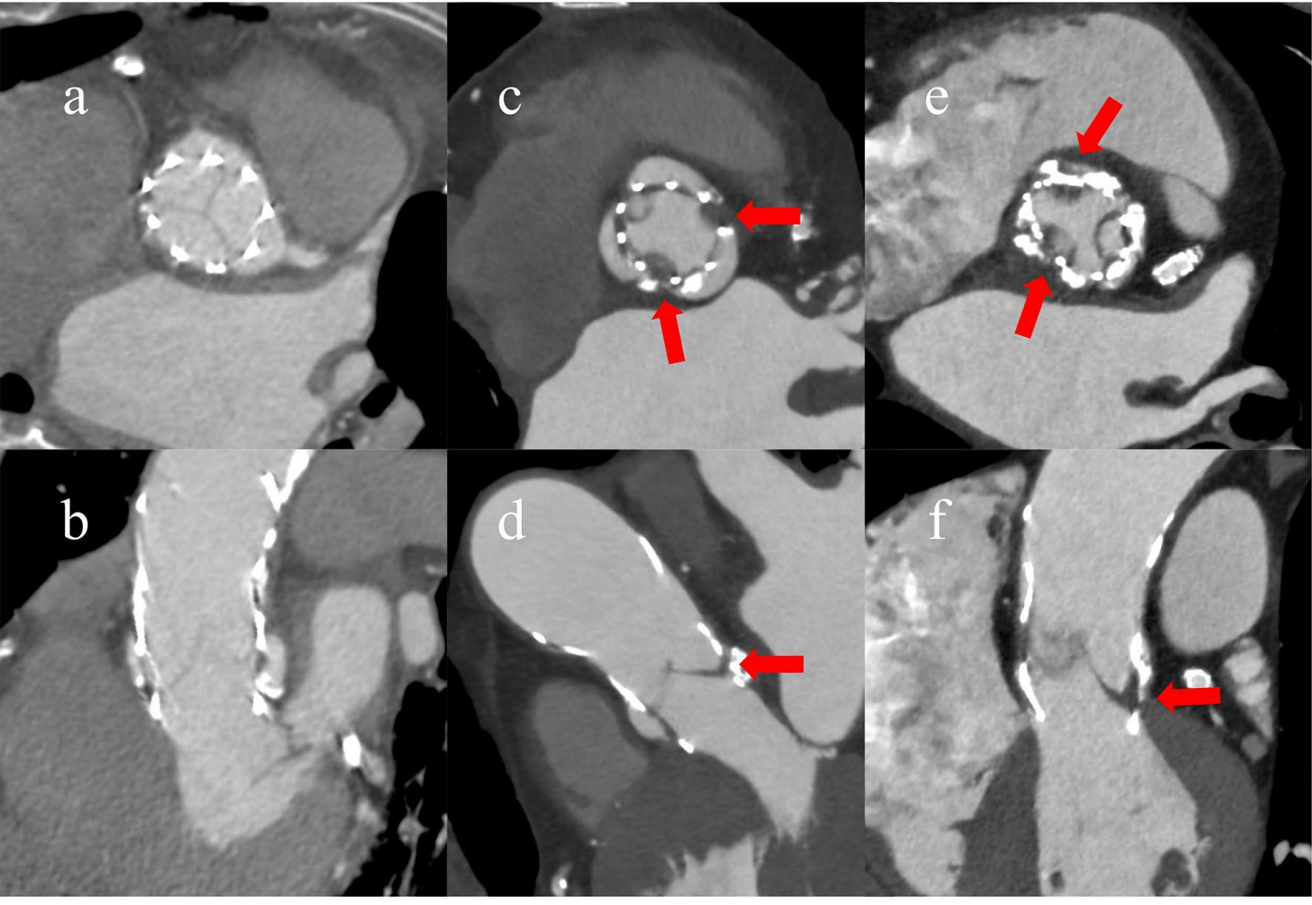
Representative cases of HALT. Contrast-enhanced CT images after transcatheter aortic valve implantation showing axial reconstructions in end-systole or mid-diastole and corresponding coronal or sagittal oblique views. **a**, **b** Views without HALT. **c**, **d** Views with grade 2 HALT. **e**, **f** Views with grade 3 HALT. Red arrows indicate the presence of HALT. *HALT* hypo-attenuated leaflet thickening, *CT* computed tomography

**Fig. 4:**
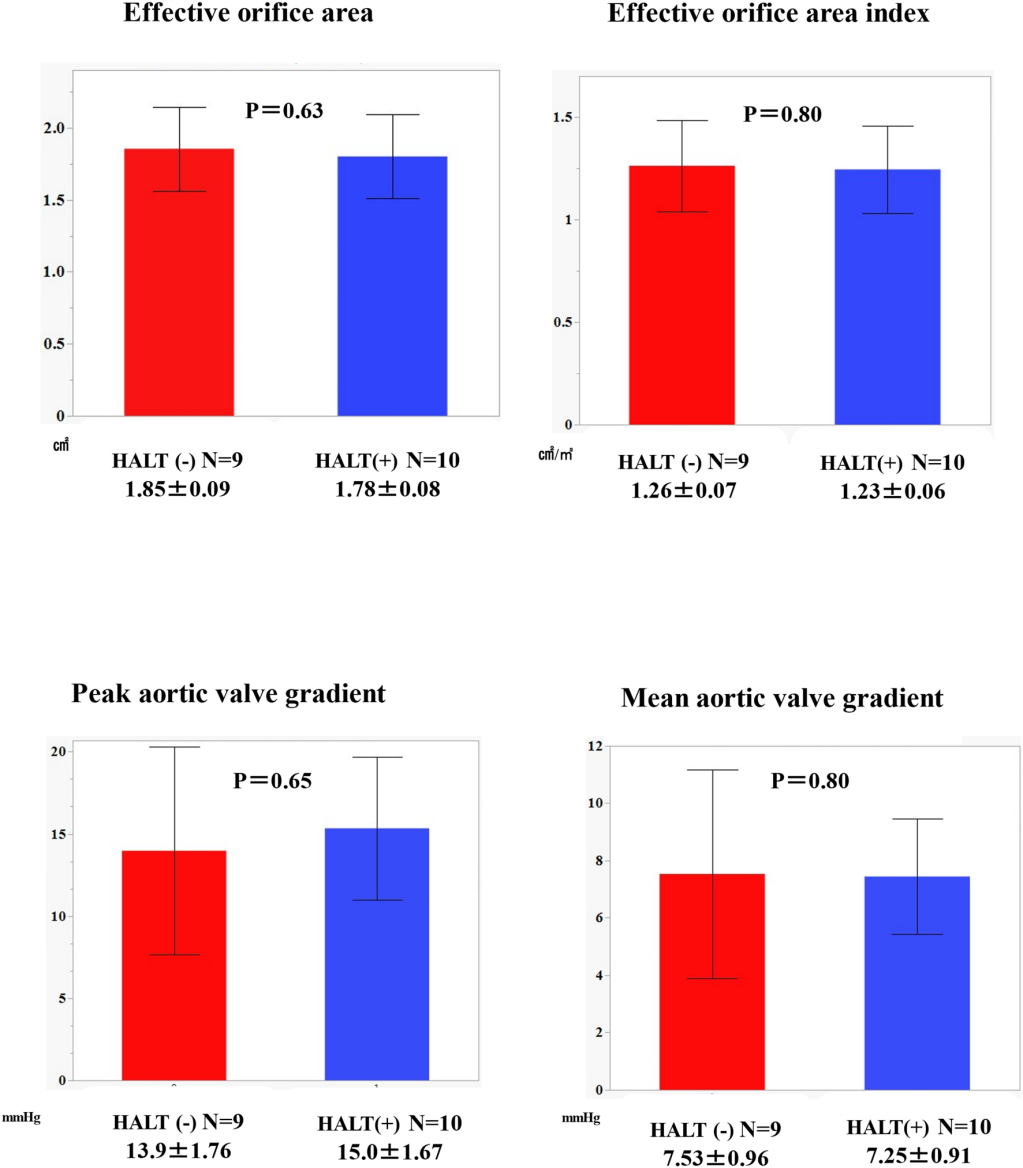
Comparisons of echocardiographic parameters 30 days after the procedure between the groups with and without HALT. No significant differences were observed in the echocardiographic parameters between the groups with and without HALT. *HALT* hypo-attenuated leaflet thickening

Contrast-enhanced CT follow-up was performed in 19 patients 30 days after the procedure, and HALT was observed in 10 (51.5%) patients (Fig. 2). The severity of HALT was grade 1 in three, grade 2 in four, and grade 3 in two patients (Fig. 3). No statistically significant differences were observed in the effective orifice area index or mean aortic valve gradient between the groups with and without HALT (Fig. 4). Similarly, peak and mean aortic valve gradients did not differ significantly between the groups with and without HALT. In the HALT group, the mean count of recaptures was 0.60 ± 0.32, whereas in the group without HALT, the mean count of recaptures was 1.00 ± 0.34.

At discharge, one patient with HALT did not receive antiplatelet or anticoagulation therapy (Table 5).

**Table 5:** Antiplatelet and anticoagulation therapies at discharge

Medication	Total cohort *N* = 32	HALT (−) *N* = 9	HALT (+) *N* = 10
Single-antiplatelet therapy	21 (66)	7 (78)	6 (60)
Dual-antiplatelet therapy	2 (6)	0 (0)	1 (10)
Anticoagulation therapy alone	7 (22)	2 (22)	1 (10)
Single-antiplatelet and anticoagulation therapy	2 (6)	0 (0)	0 (0)
None	1 (3)	0 (0)	1 (10)

Values are presented as *n* (%)

HALT: hypo-attenuated leaflet thickening

## Discussion

This study investigated the 30-day clinical outcomes and incidence of HALT in patients undergoing TAVI with the NAVITOR system. The results indicated high feasibility and safety of the NAVITOR system, as evidenced by the high rate of technical success, procedural success, device success, and early safety. The rate of permanent pacemaker implantation was as low as 3%, whereas the high incidence of HALT was observed 30 days after the procedure.

The need for pacemaker implantation after TAVI varies significantly based on the device type and the patient background, with reported rates ranging from 2 to 36% [13, 14]. The rate of pacemaker implantation after the procedure has been reported between 14.7 and 26.7% in the self-expanding Evolut™ (Medtronic, Minneapolis, MN, USA) series [15]. In our study, the rate of pacemaker implantation was lower than that reported in previous studies utilising the NAVITOR system [5, 6, 16]. One reason contributing to this lower rate could be the absence of a right bundle branch block in pre-TAVI assessments, indicating a lower baseline risk [17]. Although the length of the MS is a risk factor for pacemaker implantation, our study found no significant difference in MS length compared to other studies [18]. The reduced pacemaker implantation rate might be attributed to preoperative evaluations of MS length and subsequent adjustments to implantation position based on these measurements. In this study, the high positioning of the valve was targeted without using the distal opening method. The distal opening method involves initiating valve expansion deeper within the left ventricle (7–8 mm), partially opening the valve, and then pulling the entire device to the target depth (3–5 mm). Once optimal positioning and sufficient valve expansion are achieved, the position is maintained, and valve deployment is completed. Instead of this approach, the conventional cusp-overlap method was employed, achieving a mean implant depth of 3.1 mm at the non-coronary cusp. This successful high positioning likely contributed to avoiding the need for pacemaker implantation [7, 19]. The mean length of MS exceeded the implant depth of the non-coronary cusp (1.75 ± 2.0 mm), and successful placement above the MS length was achieved in 27 (84.5%) patients. Particular attention must be paid to preventing non-uniform expansion during the implantation of self-expanding valves. To prevent this, we first assess whether non-uniform expansion is occurring. Prior to full valve release, multi-directional fluoroscopic evaluation is performed to detect under-expansion. If non-uniform expansion is identified, the initial step is to consider recapturing and redeploying the valve, or replacing it with a new one. Additional pre-dilation with a larger balloon is then considered. Finally, following valve deployment, balloon post-dilation is evaluated. In our study, two (6.2%) of the 14 patients who underwent recapture were due to the assessment of non-uniform expansion.

Previous studies have reported that the PORTICO valve, the predecessor of the NAVITOR, achieves a sufficient valve effective orifice area with a minimal valve gradient compared to other commercially available valves [5]. The large, open cells and intra-annular design of the PORTICO valve stent frame contribute to increased haemodynamic stability during deployment and improved access to the coronary arteries post-deployment. These features have been retained in the NAVITOR valve, along with an active outer fabric cuff designed to reduce the risk of PVL by ensuring close integration with the native valve [4]. In this study, echocardiography at 30 days post-TAVI showed no moderate or severe aortic regurgitation due to PVL, with most instances classified as none or trivial, indicating favourable outcomes. These results are comparable to those seen with self-expanding valves with a supra-annular leaflet position and balloon-expandable valves [5, 16]. In addition, minimal pressure gradients and adequate valve expansion were observed in most patients, aligning with previous NAVITOR studies. These results suggest performance comparable to self-expanding valves with supra-annular leaflets, with numerically superior outcomes when compared to balloon-expandable valves.

In this study, follow-up contrast-enhanced CT was performed on 19 (59%) patients at 30 days post-TAVI, detecting HALT in 10 (52.6%) patients. Notably, this incidence was higher compared to previous reports, where HALT rates, depending on device type and patient characteristics, ranged from 7 to 15% [20, 21]. Previous studies have demonstrated that transcatheter prosthetic leaflets undergo significant stress and microtrauma during both valve delivery and deployment [11]. First, the TAVI prosthesis undergoes crimping for delivery through a small arterial sheath, which may result in irregular leaflet surfaces, microfilament damage, and reduced integrity of the pericardial leaflets [22]. In addition, valve leaflets are exposed to further damage during ballooning through direct trauma from forceful valve expansion [23]. In this study, HALT was observed in two cases that required post-dilatation with balloon aortic valvuloplasty. For repositionable valves, the process of recapturing may also lead to leaflet abrasions and further damage. In this study, the group with HALT had fewer recaptures than the group without HALT, indicating that recapture was not a significant contributing factor. Recent studies have revealed that the development of HALT is associated with neo-sinus [24]. The deployment of the TAVI prosthesis creates two distinct periprosthetic spaces: the native aortic sinus and the neo-sinus, defined as the space between the prosthesis frame and its leaflets. In vitro flow modelling comparing intra-annular valves to supra-annular valves showed that intra-annular valves tend to have larger neo-sinuses and areas of flow stagnation, particularly at the base of the prosthesis leaflets, where HALT typically forms. Previous studies evaluating HALT on CT scans 30 days post-TAVI have shown that intra-annular balloon-expandable valves exhibit a higher incidence of HALT than supra-annular self-expanding valves [20]. Therefore, the intra-annular valve design of the NAVITOR valve may contribute to a higher incidence of HALT. One of the key innovations in the NAVITOR valve, compared to the Portico valve, is the NaviSeal cuff, which encases the lower outer portion of the valve to mitigate PVL. The NaviSeal is engineered to flexibly adapt to cardiac motion and leaflet movement, effectively reducing PVL. However, it may be prone to deformation when subjected to calcific pressure, which could restrict leaflet motion and potentially contribute to the development of HALT. Although these novel findings suggest the significance of fluid haemodynamics in HALT pathogenesis, further validation in larger and more diverse cohorts is necessary.

Comorbidities, including advanced age, cancer, chronic kidney disease, diabetes, and inflammatory conditions, are associated with hypercoagulability [25, 26]. Whether a more aggressive antithrombotic strategy can reduce the incidence of HALT is currently a topic of ongoing debate. Recent guidelines recommend the selective use of oral anticoagulants for patients with confirmed HALT and elevated gradients (Class IIa recommendation, Level of Evidence B) [3]. In this study, all cases of HALT were asymptomatic, with no events such as stroke, transient ischaemic attack, or myocardial infarction occurring within 30 days post-procedure, classifying these cases as subclinical leaflet thrombosis. Haemodynamic assessment through echocardiography 30 days post-TAVI showed that patients with HALT achieved a sufficient effective orifice area index, and no cases of moderate or severe aortic regurgitation were observed. Although HALT can sometimes regress spontaneously, it has been reported to increase in many cases [26]. Previous studies have suggested that HALT does not affect valve haemodynamics up to 1 year post-procedure; however, the long-term significance remains unclear [20]. Therefore, careful monitoring for subclinical valve thrombosis via echocardiography is recommended, and contrast-enhanced CT scans should be performed during follow-up when feasible.

This study has several limitations. First, the small sample size was small, which may affect the generalizability of the findings. Second, the study utilised only a single device, limiting comparisons with other TAVI systems. Third, in this study, implant depth was assessed by 2D angiography whereas MS length was determined by 3D CT. Post-TAVI CT would likely provide a more accurate and reproducible analysis of the relationship between MS length and implant depth. However, since CT imaging was not performed in all cases following TAVI, its accuracy has not been fully assessed. Fourth, since CT scans were not performed in all patients after TAVI, there is a possibility that selection bias may have occurred.

Finally, the follow-up period was relatively short, which may not capture long-term outcomes and complications. Future studies should aim to increase the sample size, compare different devices, and extend the follow-up periods.

## Conclusion

The NAVITOR system for TAVI demonstrated favourable 30-day outcomes, with high technical success and safety rates. However, a notable incidence of HALT was observed, highlighting the need for careful follow-up. Future studies with extended follow-up periods are necessary to further evaluate the long-term clinical effectiveness of the NAVITOR system.

## Data Availability

All relevant data supporting the conclusions of this study are included in this article.
